# The efficacy of various irrigation techniques on the removal of double antibiotic paste from simulated immature roots and the amount of apically extruded debris

**DOI:** 10.1038/s41405-023-00183-3

**Published:** 2024-01-05

**Authors:** Shaimaa Nasr El-Din, Reham Hassan

**Affiliations:** 1 Faculty of Dentistry, The Egyptian Russian University, Badr, Egypt; 2 Faculty of Dentistry, The Egyptian Russian University, Badr city, Egypt

**Keywords:** Endodontic files, Root canal treatment

## Abstract

**Objective:**

This study evaluated the effect of the XP-Endo Finisher (XPF), passive ultrasonic irrigation (PUI) and conventional irrigation using side-vented needle (SVN) on the amount of apically extruded debris and canal cleanliness following the removal of double antibiotic paste (DAP) from immature root canal models.

**Material and methods:**

Forty-eight extracted mandibular premolars were drilled using peeso drills to simulate immature apices. The canals were filled with DAP and were randomly assigned into 3 groups according to the DAP removal method: XPF, PUI, and SVN (*n* = 16). The amount of extruded debris was assessed with an analytical balance then roots were split longitudinally and imaged using stereomicroscope to evaluate the residual medicament. Data were statistically analyzed using Kruskal-Wallis and Dunn’s test. Spearman’s correlation coefficient was used to determine significant correlation between extruded debris and the residual DAP scores.

**Results:**

There was no significant difference between debris extrusion values for all groups (*P* value 0.237). For canal cleanliness, the amount of remaining DAP was significantly lower in the XPF and PUI compared to SVN (*P* value < 0.001). A non-significant positive (direct) correlation was found between the amounts of apically extruded debris and residual DAP scores (*P* value 0.087).

**Conclusion:**

XPF and PUI were associated with better canal cleanliness during removal of DAP, no difference could be found between the three irrigation techniques regarding the debris extrusion.

## Introduction

Regenerative endodontic therapy (RET) is an exciting and emerging field to manage immature permanent teeth with necrotic pulps. Clinical trials have shown that RET can improve healing of the periapical infections as well as stimulate the apical development and root maturation of immature teeth [1–3]. A primary essential step in RET is to disinfect the root canal system, followed by recruitment of mesenchymal stem cells through induction of bleeding, and placement of a coronal barrier and restoration [4]. Disinfection is mainly carried out by the use of irrigants and the placement of intracanal medicaments as mechanical instrumentation is being avoided in these teeth to prevent further weakening of the fragile root canal walls [5].

Antibiotic combinations have been recommended for effective disinfection of root canal systems in RET [6].

The application and removal of intracanal medicaments requires meticulous care as immature teeth with open apices are more susceptible to apical extrusion of the intracanal medication during the placement and debris extrusion during the removal procedure, due to their wider apical diameters which may lead to inflammation, flare-up and postoperative pain [7]. Furthermore, it may evoke direct toxic effects on the stem cells in periapical tissues which are crucial for the success of the treatment [8]. Thus, any antibiotic paste remnants should be removed completely before the induction of bleeding in the second visit.

Several techniques have been recommended for efficient removal of intracanal medicaments; irrigant agitation by passive ultrasonic irrigation (PUI) is the most commonly used method [9]. PUI energy is transmitted from a file or smooth wire to the irrigant by means of ultrasonic waves that produce cavitation and acoustic streaming, responsible for enhanced cleanliness [10].

Another instrument that can be used for agitation is the XP-Endo Finisher (XPF), size 25, 0.00 taper (FKG, Dentaire SA, La Chaux-de-Fonds, Switzerland) which is made from a Max-wire alloy, XPF is quite straight when in M phase (martensitic) at room temperature, while it transform to a curved shape at body temperature as a phase transformation to A phase (austenitic) and expands up to 6 mm in diameter to clean highly complex morphologies and difficult to reach areas [11]. It has been demonstrated that this instrument has the ability to improve the removal of debris and Ca(OH)_2_ in curved and oval canals [12], improve the removal of root canal filling materials [13], reduce the bacterial load in root canals [14] and improve agitation of the irrigants [15].

Because of the considerable heterogeneity in the methodologies, including types of root canal system, types of intracanal medication, irrigation times, irrigation solutions, and their concentration and outcome measurements, the outcomes of in vitro studies comparing the effectiveness of these activated irrigation for removing intracanal medication from root canals were conflict [16].

While few studies evaluated the removal of DAP [17, 18], none up to our knowledge has measured the amount of apically extruded debris and correlate it to the cleanliness of the root canal in immature teeth. This study was therefore conducted in order to compare the efficacy of XPF, PUI and conventional irrigation using side-vented needles (SVN) with regard to the cleanliness of the canal and the amount of apically extruded debris following the removal of DAP from immature root canals. The null hypotheses tested were that there would be no differences among the irrigation activation techniques in the tested outcomes namely canal cleanliness and apical extruded debris after the removal procedure.

## Materials and methods

### Sample size calculation

Sample size calculation was done based on a primary outcome of cleaning efficacy among the 3 study groups. According to Turkaydin et al. [19] mean values for the amount of remaining triple antibiotic paste on the apical root section for conventional syringe, PUI and XPF groups were 2.30, 2.55 and 0.85, respectively using a standard deviation of (0.73) within each group. Using alpha (α) level of .05 and study power=85%; the minimum estimated sample size was (16) per group giving a total of (48) specimens. Sample size calculation was performed using IBM® SPSS® Sample Power® Release 3.0.1.

### Sample selection

Ethical approval was obtained from the ethical committee, Faculty of Dentistry, Minia University, Egypt (Registration no 59/311). Forty-eight, straight, single-rooted human mandibular premolars freshly extracted for orthodontic or periodontal reasons, were collected from the Department of Oral and Maxillofacial surgery, Faculty of Dentistry, Minia University, Egypt. Each subject signed an informed consent in which they permitted the researcher to use their tooth. Soft tissues and calculus were removed mechanically from the root surfaces with a periodontal scaler. Buccolingual and mesiodistal radiographs were taken to evaluate the root canal anatomy. Teeth with curved canals, cracks, resorptive defects or previous endodontic treatment were excluded.

### Immature teeth model

Teeth were disinfected by immersion in 5.2% sodium hypochlorite (NaOCl; Clorox, HC Egyptian Company, Cairo, Egypt) for 1 h. Decronation was done using a tapered diamond stone mounted on low-speed hand piece under water coolant to obtain a standardized root length of 15 mm. Root canal patency was checked and the working length (WL) was determined using K-files #10 (Dentsply Sirona, York, PA, USA) placed until it became visible at the apical foramen and subtracting 1 mm from that length of the file. To simulate an immature tooth with open apex, teeth were instrumented using peeso drills (MANI Inc, Toshigi-Ken, Japan) from size 1–4 to create an apical opening of 1.3 mm in diameter and simulate an open/immature apex. Root canals were irrigated with 2 mL 1.5% NaOCl using a 30 gauge(G) SVN (Maxi-i-probe, Dentsply, Rinn, IL, USA) at every instrument change. Final irrigation was completed using 5 mL 17% EDTA for 1 min and 5 mL 1.5% NaOCl for 1 min followed by the final rinse with 5 mL distilled water. Finally, canals were dried using sterile paper points (Dentsply Maillefer, Ballaigues, Switzerland).

DAP was prepared by grinding an equal number of tablets of 500 mg metronidazole (Flagyl; Aventis, Cairo, Egypt) and 500 mg ciprofloxacin (Ciprofar; pharco, Alexandria, Egypt). The powder (1:1) was then mixed with distilled water; final paste concentration was 1 mg/mL based on recommendation of the American Association of Endodontists [20]. The paste was applied to the canal spaces with 3 cm plastic syringe. Cotton pellets were placed over the canal orifices, and a temporary sealing material (Cavit G; 3 M ESPE, Seefeld, Germany) was used to seal the access cavities. Apical openings were sealed with sticky wax then all samples were stored at 37 °C in 100% relative humidity. After 2 weeks, cotton pellets, temporary filling and the sticky wax were removed.

### Prototype for collection of extruded debris

The experimental model described by Myers and Montgomery [21] was used in this study. The stoppers of Eppendorf tubes were removed, and tubes were numbered and weighed using an analytical balance (Adam Equipment Co. Ltd, MK10 0BD. UK) with an accuracy of 10^−4^ g. Three consecutive weights were obtained for each tube, and the mean value was considered to be its initial weight (W1). A hole was created with a heated instrument in the center of the rubber stopper of each tube, and then each tooth was inserted under pressure into the rubber stopper and fixed at the cemento-enamel junction using cyanoacrylate glue. A 27 G needle was placed alongside the cover as a drainage cannula to equalize the internal and external air pressure. Next, each unit including the stopper, tooth and needle, was fixed to its Eppendorf tube. The tubes were fitted into vials to hold the device during preparation. The operator was blinded from seeing the root apex during the removal procedure by covering the vial with aluminum foil. Samples were randomly divided using (https://www.randomizer.org) into three equal experimental groups (*n* = 16) as follows;

#### XP-Endo Finisher group

The specimens were rinsed with 10 mL 1.5% NaOCl using a syringe and 30 G SVN (Maxi-i-probe, Dentsply, Rinn, IL, USA) placed 1 mm from the WL with a flow rate of approximately 5 mL/min. XPF file (size 25, 0.00 taper) (FKG, LaChaux-de-faund, Switzerland) was used with an endodontic motor (X‐Smart, Dentsply‐Maillefer, Ballaigues, Switzerland) at 800 rpm and 1 Ncm torque according to the manufacturer’s instructions [11]. The file was inserted 1 mm shorter than the WL which was adjusted using the plastic tube to fix the rubber stopper and operated for 60 s using slow and gentle 7–8 mm lengthwise in‐and‐out movements. Each file was used for one canal in order to prevent interfering with the debris extrusion results.

#### Passive ultrasonic irrigation group

PUI was performed using IrriSafe ultrasonic tip (size 25, 0.00 taper) (Acteon, France) mounted on an ultrasonic system (Newtron P5; Satelec, Acteon group, France) in an endo power setting at 5^th^ power. The root canal of each specimen was rinsed with 10 mL 1.5% NaOCl using 30 G SVN (Maxi-i-probe, Dentsply, Rinn, IL, USA) placed 1 mm from the WL with a flow rate of approximately 5- mL/min. Then, the IrriSafe tip was inserted into the canal 1 mm short of the WL, and the irrigant was ultrasonically activated for 20 s. This sequence was repeated two more times; the tip was kept as centered as possible to minimize contact with the canal walls, as any contact with the canal wall could dampen the oscillatory motion of the tip [22]. Each tip was used for one canal in order to prevent interfering with the debris extrusion results.

#### Needle irrigation group

Root canals were rinsed with 10 mL 1.5% NaOCl using a 5- mL disposable plastic syringe and 30 G SVN (Maxi-i-probe, Dentsply, Rinn, IL, USA). The needle was inserted passively up to 1 mm short of the WL with a flow rate of approximately 5 mL/min. During irrigation, the needle was constantly moved up and down within the apical third.

The irrigants temperature, flow rate, volume and activation time were standardized for all groups. Rubber stoppers were placed on the XPF, IrriSafe ultrasonic tip and the needle at the required length to ensure length control. In all groups, a final flush using 2 mL 17% EDTA was used. NaOCl solution used in all groups was warmed to 37 °C prior to the application to allow the XPF to work optimally at the austenite phase.

### Assessment of debris extrusion

On completion of the intracanal removal procedure, the stoppers, needles, and teeth were gently removed from their tubes and vials, then the debris adhered to the root surface were collected by washing off the apex with 1 mL of distilled water into the tube. Eppendorf tubes were stored in an incubator at 37 °C for 15 days to evaporate the moisture before weighing the dried debris. Weighing procedure was carried out again using the same balance and three consecutive weights were obtained for each tube, then the mean was calculated. These measurements considered to be the weight of the tube plus the debris (W2). The dried weight of the extruded debris was calculated by subtracting the weight of the empty tube from that of the tube containing debris (W2-W1).

### Assessment of canal cleanliness

**Fig. 1:**
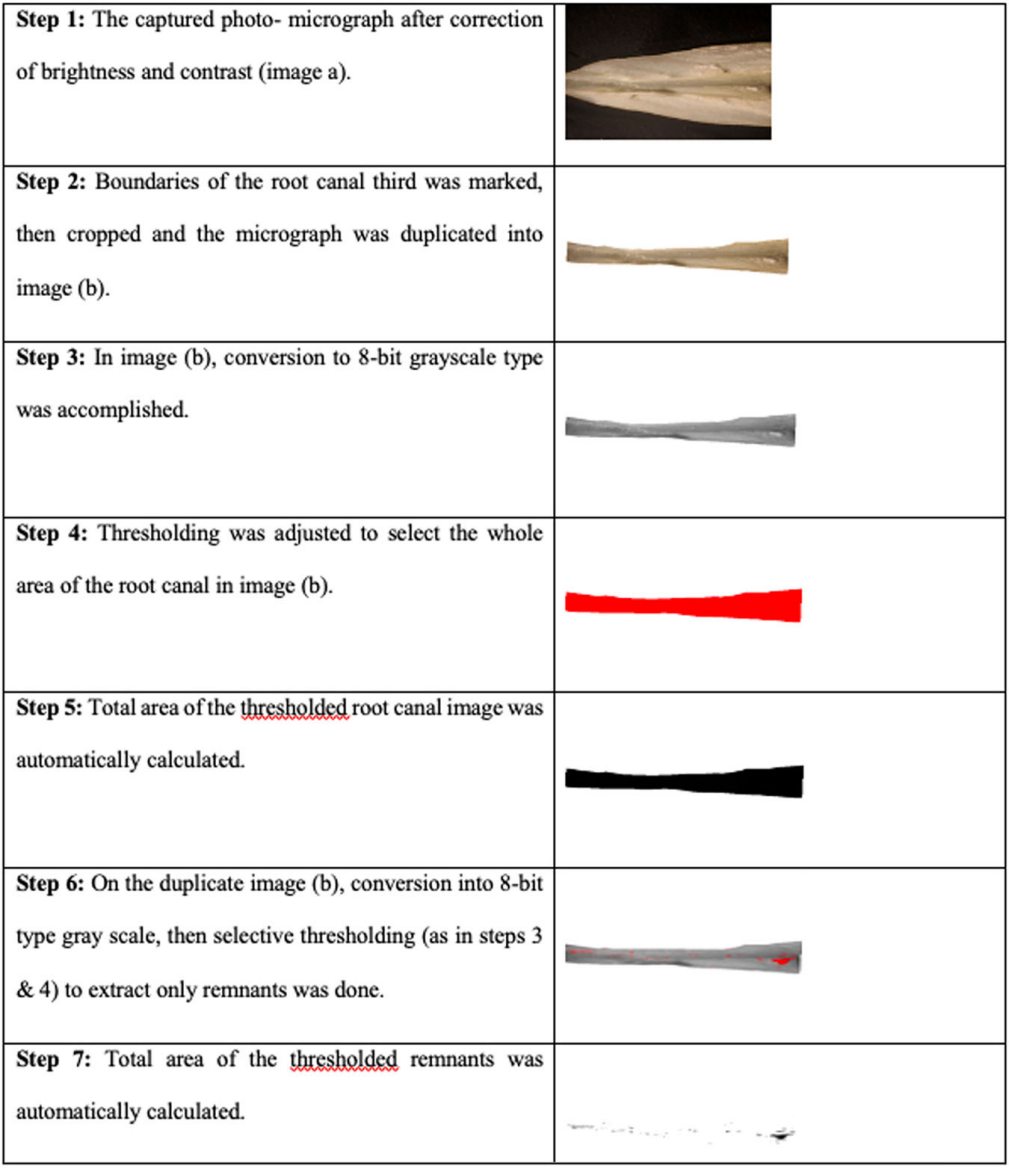
Steps of quantitative evaluation of the area of remaining DAP in a specimen (stereomicroscope images mag. x10). Area fraction of root canal remnants was estimated mathematically by dividing (total area of remnants within the root canal / total area of root canal) x100.

Longitudinal grooves were prepared on the buccal and the lingual surfaces of each sample with a thin diamond bur used in high speed hand-piece, shallow grooves were made keeping the dentin surrounding the root canal intact. A Hammer and chisel were used to split each tooth into two longitudinal halves. Photographs were captured at the magnification of 10x using digital camera (EOS 650D, Canon, Japan) which was mounted on a light stereomicroscope (BX60, Olympus, Japan) at the Precision Measurement Unit, Oral Pathology Department, Faculty of Dentistry, Ain Shams University, Egypt. Images were given random numbers to prevent the identification by the assessor. Each Image was analyzed using Image J software program (Image J 1.41 A, National Institute of Health, USA). First, the overall area of the canal space visualized in the images was delimited and this measurement corresponded to 100%. Next, the area of the remaining DAP was measured and the percentage of remaining paste was calculated from the ratio between the area of the remaining DAP and the canal spaces (Fig. 1). The percentage for each specimen was converted into a scoring system following the protocol previously described by Küçükkaya et al. [23].

A scoring system described by Turkaydin et al. [19] was used to evaluate the presence of the remaining DAP on the canal wall;

1. Score 0: Absence of any residue.
2. Score 1: Small amount of residues (up to 20% of the surface covered).
3. Score 2: Moderate amount of residues (20–60% of the surface covered).
4. Score 3: Large amount of residues (more than 60% the surface covered).

All root canals were prepared, by the same experienced operator (S.N) to reduce inter-operator variability, while measurements were done by an examiner who was blinded to the study groups (R.H), Intraobserver reproducibility was verified, based on intraclass correlation coefficients (ICCs).

### Statistical analysis

Numerical data were explored for normality using (Kolmogorov-Smirnov and Shapiro-Wilk tests). All data showed non-normal (non-parametric) distribution. Data were presented as median, range, mean and standard deviation (SD) values. Kruskal-Wallis test was used to compare between the three irrigation techniques. Dunn’s test was used for pair-wise comparisons when Kruskal-Wallis test is significant. Spearman’s correlation coefficient was used to determine significant correlation between extruded debris and remaining debris scores. The significance level was set at *P* ≤ 0.05. Statistical analysis was performed with IBM SPSS Statistics for Windows, Version 23.0. Armonk, NY: IBM Corp.

## Results

### Apical extruded debris

**Fig. 2:**
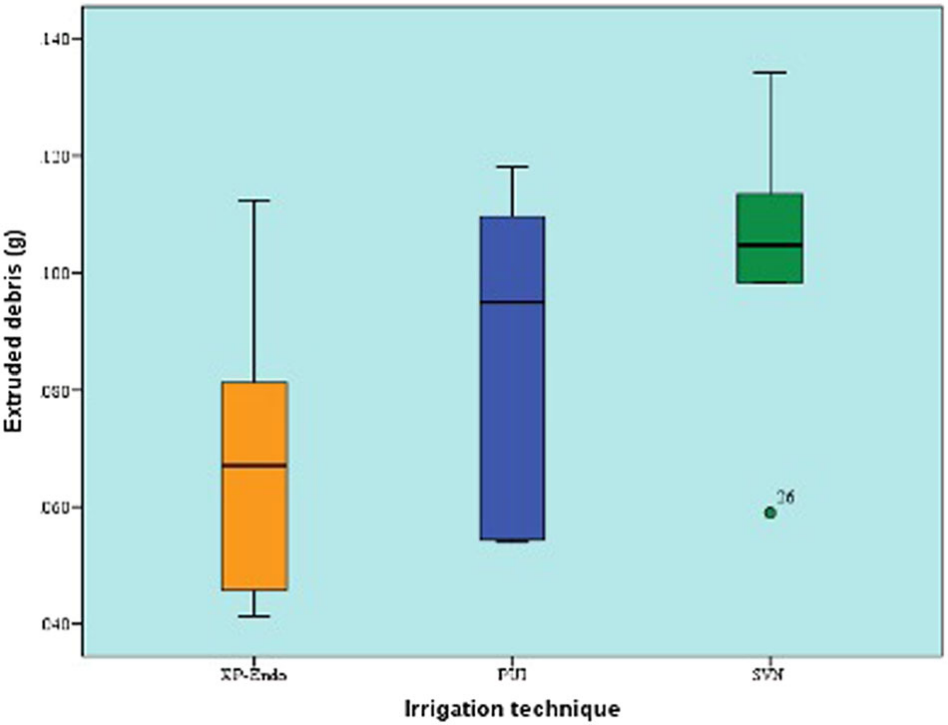
Box plot of the amount of apically extruded debris. Box plot representing median and range values of the amounts of apically extruded debris with different irrigation techniques after the removal of DAP (Circle represents outlier).

Figure 2 shows median and range values of the amount of apically extruded debris of XPF, PUI, SVN after the removal of DAP. All techniques showed considerable amount of apically extruded debris. However, no statistically significant difference could be found between the three irrigation techniques (*P* value = 0.237, Effect size = 0.073).

### Canal cleanliness

**Fig. 3:**
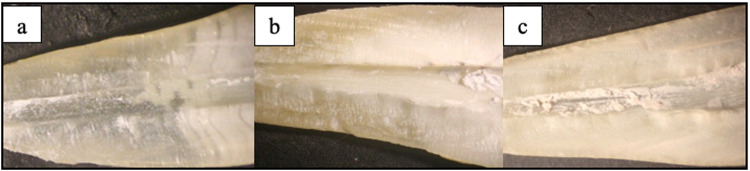
Stereomicroscopic image of the root canal wall. Stereomicroscopic image of the remaining DAP on the root canal walls after removal with (**a**) XPF group. (**b**) PUI group. (**c**) SVN group.

The level of intraobserver agreement was excellent (Supplemental File 1). Median, range, mean and standard deviation (SD) values of the remaining debris scores of XPF, PUI and SVN after the removal of DAP are presented in (Table 1). None of the experimental groups removed DAP completely from the root canals (Fig. 3). There was a statistically significant difference between debris scores of remaining DAP after the removal with irrigation techniques (*P* value < 0.001, Effect size = 0.772). Pair-wise comparisons between the techniques revealed that; both the XPF and PUI removed significantly more DAP than SVN. However, there was no statistically significant difference between the XPF and PUI groups. SVN was the least efficient method in removing DAP.

**Table 1:** Descriptive statistics and results of Kruskal-Wallis test for comparison between the remaining debris scores of DAP in root canal wall after the removal with three irrigation techniques.

Irrigation technique	Debris score of DAP	
	Median (Range)	Mean (SD)
XP-Endo	1 (1–1)^B^	1 (0)
PUI	1 (0 – 2)^B^	1.1 (0.57)
SVN	3 (2–3)^A^	2.7 (0.48)
*P* value	<0.001*	
Effect size (Eta Squared)	0.772	

*Significant at *P* ≤ 0.05, Different superscripts in the same column are statistically significantly different.

### Correlation between debris score and apically extruded debris

**Fig. 4:**
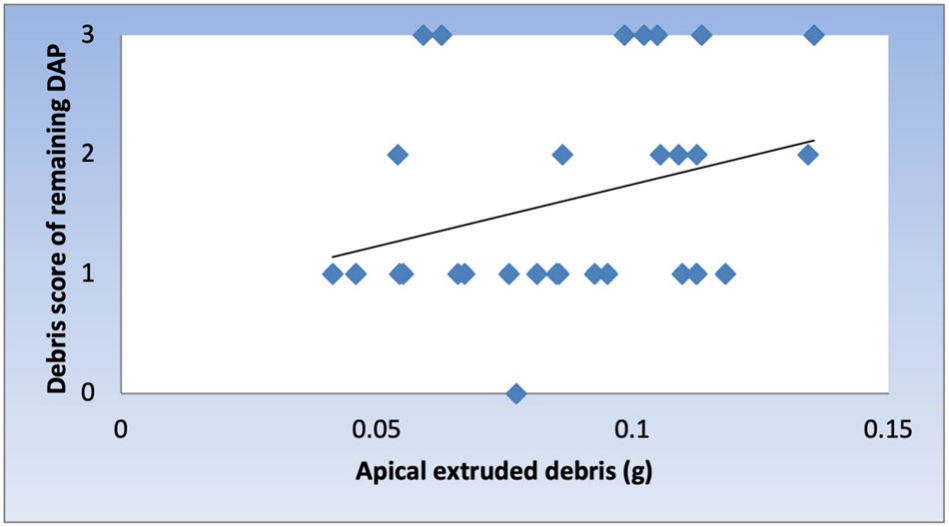
Scatter diagram of Spearman’s correlation coefficient. Scatter diagram representing direct correlation between the amount of apically extruded debris and debris scores of remaining DAP.

Spearman’s correlation coefficient showed a direct (positive) correlation between the amount of apically extruded debris and debris scores of remaining DAP without statistically significant difference (*ρ* = 0.318, *P* value = 0.087) (Fig. 4).

## Discussion

Recently, DAP has been emerging as an intracanal medicament of choice in endodontic regeneration due to its significant antibacterial properties against different endodontic pathogens [24], as well as its minimum tooth discoloration potential compared to TAP [25]. Moreover, Recent studies proposed that DAP has superior antimicrobial properties than Ca(OH)_2_ [24, 26].

This study highlights the importance of correlating between complete removal of intracanal medications and the amount of extruded debris apically during RET in a trial to lower the cytotoxic effect of intracanal medicaments against stem cells pivotal for the success of the treatment.

An immature model was implemented to mimic the clinical scenario with wide root canal and apical foramen, which was achieved by the use of peeso reamers that facilitated the standardization of the dimensions of root canal spaces [27]. Trevino et al. [28] suggested using LSX instrument to the size of 130 for creating immature apex. Since a peeso reamer size 4 corresponds to size 130, it was used to create the immature apex model in this study.

The application period of intracanal medicaments varies widely between published studies, where some cases showed success after 1 week in RET [29]. Er et al. [30] used TAP for intracanal dressing for up to 12 weeks in root canal treatment. According to the AAE recommendation for RET [20]; time interval between first and second appointment varies from one to four weeks, thus the chosen time period for this study was 2 weeks.

For canal cleanliness, antibiotic pastes were previously removed using various irrigating solutions such as NaOCl, EDTA, and sterile saline [31]. The combination of EDTA and NaOCl irrigants improves the removal efficiency [32]. In this study, final EDTA irrigation was performed to simulate the actual clinical scenario during endodontic regeneration. The concentration of the irrigants used was adopted from the AAE [20] and the European Society of Endodontology (ESE) position statement for revitalization procedures [5]. On the other hand, in previous apical extrusion studies [33] distilled water was used as an irrigant as the use of NaOCl might cause precipitation of sodium crystals which interfere with the amount of apically extruded debris. In the present study, it seemed logical to use the routinely used irrigation solutions to better reflect the clinical situation. Keeping in consideration, to be able to determine the correlation between canal cleanliness and apical extruded debris, same teeth with same irrigant type, volumes and activation time had to be used for both tests.

For standardization, one minute of activation was carried out for both the XPF and PUI. Also, all instruments were kept 1 mm short from the apical foramen as it has been observed to cause less dentinal debris and irrigant extrusion [21].

One of the limitations of the present study was the inability to simulate the intracanal temperature, While the XPF instruments can function properly only at body temperature (martensitic phase) [34], yet placing the samples in hot water bath following other studies protocol [35] would have an impact on the solubility of the DAP and would disturb the debris extrusion measurement. To overcome this shortcoming in the present study, all irrigating solution was preheated at 37 °C to mimic body temperature.

Stereomicroscope images were analyzed using Image J software program that calculated the percentage of the remaining paste inside root canal of each specimen [23] which were further converted into a score, this methodology was preferred over the scoring method used in previous studies [19, 36] as it provides a quantitative analysis and prevents observer bias.

Previous studies reported that no existing irrigation solutions or activation method can render the main canal completely free of residual medication [10, 18, 19]. These observations are similar to the findings of the present study, none of the tested methods were able to completely remove DAP from immature root canals. Needle irrigation is the traditional method used to deliver different irrigation solutions into the root canal space. Our findings support previous reports [18] were SVN showed limited cleaning ability while the use of XPF and PUI improved the removal of DAP significantly compared to the SVN, without significant difference between both activation methods [16, 36–38]. Thus, the null hypothesis regarding canal cleanliness was rejected.

The PUI have demonstrated its surplus effect on medication removal [18, 39] which could be attributed to the acoustic energy delivered by the ultrasonic tip, activating the irrigation solution and creating cavitation bubbles and heat generation of the irrigant [40]. Our results disagree with that of Turkaydin et al. [19] who found no significant difference between PUI and standard needle irrigation in the removal of TAP. This conflicting result could be explained by the different methodology employed, where they applied the intracanal medication for 1 month which could have negatively affected the removal efficacy [41]. Moreover, root canals were ultrasonically irrigated using a continuous flow and operated in 2 cycles of 1 min as opposed to intermitted flow at 3 cycles of 20 s each used in the present study.

Our results showed that the use of XPF increased the cleanliness of root canals, which is in agreement with previous reports [27, 41, 42]. XPF possess an added actual physical removal capability of medication in addition to the dissolution ability of the irrigants activated with XPF. According to the manufacturer, XPF allows mechanical cleaning of canal areas previously impossible to reach with standard instruments [11]. The file design is based on the shape memory principles of the NiTi alloy; where it transfer from martensitic-to-austenitic phase when it is exposed to the canal temperature which allows the file to contract and expand according to the root canal anatomy. Furthermore, a 7–8 mm vertical movement was applied in an attempt to contact the full length of the canal.

A contradicting result could be found by Donnermeyer et al. [43] were they found PUI to be significantly more effective than the XPF in the apical region. This conflict could be explained by the irrigant temperature, as 20 °C was used in their study which could have prevented the martensitic-to-austenitic transformation leading to decreased efficacy of the XPF.

The 1 min operation time suggested by the manufacturer [11], was not sufficient for the effective removal of DAP from the canal in this study; longer operation periods should be tested. Denna et al. [44] noticed that the combined use of XPF with PUI resulted in almost complete removal of Ca(OH)_2_ from root canals with the highest efficacy, further studies concerning the effectiveness of this combination in removal of DAP is required.

In the second part of this study, the volume of debris extrusion after the removal of DAP from immature teeth were measured, to our knowledge, no studies have been done to assess apical debris extrusion after the removal of DAP with XPF, PUI and SVN in immature teeth, thus direct comparison with results from previous studies could not be accomplished.

Results of this study indicated that all irrigation techniques used was associated with some level of apically extruded debris without significant differences among them; hence, the null hypothesis regarding debris extrusion was accepted. This came in line with a previous study by Dos Reis et al. [45] who showed no significant difference in the irrigant extrusion between no agitation group, ultrasonic agitation with Irrisonic, ultrasonic agitation with Irrisonic Power, mechanical agitation with Easy Clean, mechanical agitation with the XPF.

Finally, there was a non-significant positive correlation between the amount of apically extruded debris and remaining DAP debris score after the removal procedure with different irrigation techniques.

One potential limitation of our study was the inability to obtain the necessary sample size using extracted teeth with immature roots. As a result, we opted to conduct an immature model in this study to better replicate the clinical scenario. However, this model also presented a weakness in that it lacked the simulation of periodontal tissue, as the foramina were suspended in air (zero back pressure). Thus, results obtained from this in vitro model may differ from a clinical situation where the periodontium acts as a natural barrier, possibly limiting the extrusion of debris.

## Conclusions

XPF and PUI were associated with better canal cleanliness during removal of DAP, no difference could be found between the three irrigation techniques regarding the debris extrusion. Further studies are required to assess the effect of increasing the activation time of XPF and the combination of both XPF and PUI in the removal of intracanal medication.

### Supplementary information

Supplemental Information

## Data Availability

The datasets used and/or analysed during the current study are available from the corresponding author on reasonable request.
