# Supplementary material for: The efficacy of various irrigation techniques on the removal of double antibiotic paste from simulated immature roots and the amount of apically extruded debris
Source: BDJ Open. 2024 Jan 5;10:2. doi: 10.1038/s41405-023-00183-3 (PMC10770069; doi:10.1038/s41405-023-00183-3)
Supplement: Supplementary file 1 — Supplemental Information [file 41405_2023_183_MOESM1_ESM.pdf]

Supplemental File 1

Intraclass correlation coefficient for the cleaning score

	Intraclass correlation [b]	95% Confidence interval		*F* test with true value 0			
		Lower bound	Upper bound	Value	df1	df2	Sig
**Single measures**	.971[a]	.950	.985	81.420	59	59	.000
**Average measures**	.987	.971	.992	81.420	59	59	.000

Two-way random effects model in which people effects and measures effects are random.

[a] The estimator is the same, regardless of whether the interaction effect does or does not exist.

[b] Type A intraclass correlation coefficients, using the absolute agreement definition.

PAI, periapical index; df1, degrees of freedom 1; df2, degrees of freedom 2; Sig, significance
